# Choline and Choline Metabolite Patterns and Associations in Blood and Milk during Lactation in Dairy Cows

**DOI:** 10.1371/journal.pone.0103412

**Published:** 2014-08-26

**Authors:** Virginia M. Artegoitia, Jesse L. Middleton, Federico M. Harte, Shawn R. Campagna, Michael J. de Veth

**Affiliations:** 1 Department of Food Science and Technology, The University of Tennessee, Knoxville, Tennessee, United States of America; 2 Department of Chemistry, The University of Tennessee, Knoxville, Tennessee, United States of America; 3 Department of Animal Science, The University of Tennessee, Knoxville, Tennessee, United States of America; 4 Balchem Corporation, New Hampton, New York, United States of America; Medical University of South Carolina, United States of America

## Abstract

Milk and dairy products are an important source of choline, a nutrient essential for human health. Infant formula derived from bovine milk contains a number of metabolic forms of choline, all contribute to the growth and development of the newborn. At present, little is known about the factors that influence the concentrations of choline metabolites in milk. The objectives of this study were to characterize and then evaluate associations for choline and its metabolites in blood and milk through the first 37 weeks of lactation in the dairy cow. Milk and blood samples from twelve Holstein cows were collected in early, mid and late lactation and analyzed for acetylcholine, free choline, betaine, glycerophosphocholine, lysophosphatidylcholine, phosphatidylcholine, phosphocholine and sphingomyelin using hydrophilic interaction liquid chromatography-tandem mass spectrometry, and quantified using stable isotope-labeled internal standards. Total choline concentration in plasma, which was almost entirely phosphatidylcholine, increased 10-times from early to late lactation (1305 to 13,535 µmol/L). In milk, phosphocholine was the main metabolite in early lactation (492 µmol/L), which is a similar concentration to that found in human milk, however, phosphocholine concentration decreased exponentially through lactation to 43 µmol/L in late lactation. In contrast, phosphatidylcholine was the main metabolite in mid and late lactation (188 µmol/L and 659 µmol/L, respectively), with the increase through lactation positively correlated with phosphatidylcholine in plasma (*R* ^2^ = 0.78). Unlike previously reported with human milk we found no correlation between plasma free choline concentration and milk choline metabolites. The changes in pattern of phosphocholine and phosphatidylcholine in milk through lactation observed in the bovine suggests that it is possible to manufacture infant formula that more closely matches these metabolites profile in human milk.

## Introduction

**Figure 1:**
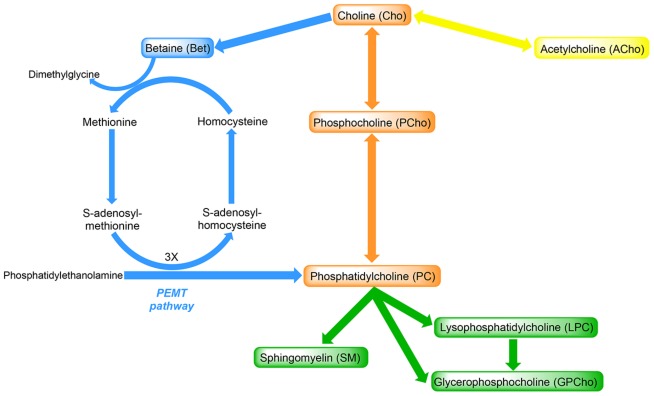
Metabolism of choline and its metabolites. The compounds shown in boxes were assayed in milk and plasma in the current study. Phosphocholine and phosphatidylcholine are formed from choline via the cytidine diphosphate (CDP) choline pathway. The formation of betaine from choline is irreversible. Betaine when oxidized will provide a methyl group to homocysteine to form methionine. Methionine is converted to S-adenosylmethionine, which is an important methyl donor. Phosphatidylcholine can be formed endogenously by methylating phosphatidylethanolamine in a three step process involving S-adenosylmethionine via the phosphatidylethanolamine *N*-methyltransferase (PEMT) pathway.

Choline is found in various forms in humans and animals [1], [2] (Figure 1). The water-soluble choline metabolites include acetylcholine (ACho), an important neurotransmitter for brain and neuromuscular function [1], betaine (Bet), an oxidative intermediate of choline which supplies a methyl group for the conversion of homocysteine to methionine [3], and glycerophosphocholine (GPCho), which like Bet acts as an organic osmolyte in cells [4]. The lipid-soluble choline-containing metabolites phosphatidylcholine (PC), sphingomyelin (SM), and lysophosphatidylcholine (LPC) are all structural components of mammalian membranes [5]. PC is essential for synthesis of VLDL, a key mechanism to export triacylglycerol from the liver [6].

Choline can be obtained from the diet [7] and from endogenous biosynthesis that predominantly occurs in the liver via the phosphatidylethanolamine *N*-methyltransferase (PEMT) pathway [8]. Although the PEMT pathway represents an important source of choline [3], dietary intake of choline is necessary because the pathway supplies insufficient choline moiety to maintain the normal function of cells, tissues and organs [7], [8]. In the case of the ruminant, however, dietary choline is extensively degraded (>80%) by rumen bacteria [9], [10] and therefore endogenous synthesis via the PEMT pathway represents a critical source of choline [11]. For the lactating dairy cow, the periparturient period is characterized by a high incidence of fatty liver disease and supplementation of rumen-protected choline has been found to reduce the extent of hepatic fatty infiltration and increase expression of genes involved in VLDL transport [12], [13].

Despite the lack of dietary-derived choline in the ruminant, milk and dairy products are an important source of choline in the human diet. One cup of retail milk (2% fat) is expected to provide approximately 40 mg choline [14], which represents nearly 10% of an adult woman's daily choline requirement [15]. In addition, bovine milk is the main ingredient used in the manufacture of infant formula [16], which is used during a period of human development where choline requirements are high for organ growth and membrane biosynthesis [17]. Studies that have compared the choline content of breast milk and infant formula have found that whilst the total choline in breast milk was relatively constant [1250–1481 µmol/L), choline in bovine-derived infant formula varied by over 7-fold (311–2270 µmol/L) [18], [19]. Further, these studies have found the proportion of the various choline metabolites in milk differs for breast milk and infant formula, which may be important for the neonate as the bioavailability of the choline metabolites can vary considerably [20]. Despite the variation in choline content and choline metabolite proportions of bovine-derived infant formula, it is not known to what extent this variation may be attributed to the milk initially harvested from cows or due to post-harvest processing.

Lactation is characterized by extensive physiological adaptations that are coordinated to provide the appropriate quantity and pattern of nutrients for milk synthesis [21]. These adaptations have been associated with changes in choline metabolism, with differences in choline metabolite concentrations during lactation reported in breast milk and porcine milk [18], [22]. However, there has not been a systematic evaluation of changes in choline metabolites in plasma and milk during lactation in dairy cows. Recently, a method based on hydrophilic interaction liquid chromatography-tandem MS (HILIC LC-MS/MS) was developed and validated to identify and quantify all of the major choline-containing compounds as well as their fatty acid composition within a phospholipid class [23], [24].

The objectives of the current study were threefold. Firstly, we sought to characterize the changes in choline and choline metabolites in blood and milk over the first 37 weeks of lactation (WOL) by sampling during early, mid and late lactation, which is indicative of periods in lactation where the physiological state differs greatly in the dairy cow. The quantification of choline output in milk will contribute to an understanding of the mammary glands' requirement for choline during lactation. In addition, changes in the different PC species were of interest as recent research has shown that PC species with long chain polyunsaturated fatty acids, primarily docosahexaenoic acid (DHA), originated from PEMT activity in the liver of rodents and humans [25], [26]. Secondly, we sought to determine whether the yield of choline and choline metabolites in milk were associated with changes in blood choline metabolite concentrations. Previously, weak correlations between blood and milk choline concentrations were found in nursing women [18]. In the present study, we had the advantage of monitoring the same animals throughout lactation, which was expected to reduce the variation and improve the likelihood of observing interrelationships. Thirdly, we sought to determine the relationship between milk choline yield and common animal performance variables (i.e. milk yield, fat and protein content, and WOL) to evaluate whether easily measurable end points could be used to predict total milk choline output.

## Materials and Methods

### Ethics Statement

Animal experimentation was approved by The University of Tennessee Institutional Animal Care and Use Committee.

### Experimental Design

Twelve multiparous Holstein cows averaging 653±55 kg (mean ± SEM) body weight were recruited at calving and managed under the same diet, without choline supplementation, throughout the study.

The diet was a total mixed ration (TMR) formulated using the National Research Council requirements [27] and were fed ad libitum at 0700 and 1900 daily (Table 1). Water was available at all times. TMR samples were collected once each month during the experiment and stored at −40°C before being analyzed for chemical composition by wet chemistry (Dairy One Cooperative, Inc., Ithaca, NY). The individual ingredients that comprised the TMR diet were analyzed for choline metabolites (Table 2).

**Table 1:** Ingredient and chemical composition of total mixed ration.

Ingredient (g/kg dry matter)^1^	
Corn silage	644
Lactating supplement^2^	270
Corn	18
Clover hay	22
Rye grass hay	28
Alfalfa hay	18

Chemical analysis (g/kg dry matter)^3^	
Crude protein	181
Neutral detergent fiber	452
Acid detergent fiber	312
NEL^4^ (Mcal/kg dry matter)	0.72

1 Dietary dry matter averaged 840 g/kg.

2 Contained per kilogram: ground corn 197 g, cottonseed 200 g, wheat mids 95 g, corn gluten feed 85 g, cottonseed hulls 75 g, citrus pulp 70 g, soybean meal 65 g, cottonseed meal 60 g, distillers grain with soluble 46 g, calcium salts of palm fatty acids 20 g, fish meal 20 g, cane molasses 20 g, ground limestone 20 g, sodium bicarbonate 10 g, urea 7 g, salt 5 g, Zn methionine 5 g. vitamin A 12665 IU, vitamin D 2760 IU, vitamin E 90 IU, selenium 0.3 mg, copper 12.9 mg, Zinc 50 mg, cobalt 0.23 mg, manganese 49 mg, iron 80 mg, iodine 0.50 mg.

3 Analysis by Dairy One Inc., Ithaca, New York.

4 Net energy for lactation.

**Table 2:** Choline and choline metabolite concentration in individual feed ingredients used to make the total mixed ration.

Ingredient	Choline and choline metabolite^1^, mg/100 g								
	ACho	Bet	Cho	LPC	PC	PCho	SM	GPCho	TC^2^
Corn silage	0.07	5.3	12.9	0.2	8.6	0.01	nd	0.26	22.1
Supplement^3^	0.12	65.2	23.1	27.6	974.7	0.75	0.39	40.02	1132.0
Corn	nd	5.0	9.8	4.0	30.9	0.08	nd	3.18	47.9
Clover hay	0.02	10.7	39.0	3.7	74.2	0.68	0.06	5.19	122.9
Rye grass hay	0.02	8.4	33.3	0.6	11.8	0.14	0.02	0.91	46.8
Alfalfa hay	0.06	16.6	43.3	4.0	3.8	0.14	nd	2.69	54.0

1 Acetylcholine (ACho), betaine (Bet), free choline (Cho), glycerophosphocholine (GPCho), lysophophatidylcholine (LPC), not detected (nd), phosphatidylcholine (PC), phosphocholine (PCho), sphingomyelin (SM), total choline (TC).

2 TC is the sum of ACho, Cho, LPC, PC, PCho, SM and GPCho.

3 See Table 1 for ingredient composition of the supplement mix.

Milk and blood samples were collected on one day each week during three periods of the lactation, early lactation (weeks 1, 2 and 3; n = 12), mid lactation (weeks 7, 10 and 13; n = 12) and late lactation (weeks 31, 34 and 37; n = 10). Cows were milked at 0600 and 1800 h daily. Milk was sampled and yield determined at both daily milkings (pm and am) of each week. One aliquot was stored at 4°C with a preservative (bronopol tablet; D&F Control System, San Ramon, CA) until analyzed for fat and true protein content (Tennessee DHIA, Inc., TN) by mid-infrared spectrophotometry (Milko-Scan, Foss Electric, Hillerød, Denmark). A second aliquot of milk was stored at −40°C for choline metabolite analysis. Blood samples were taken after the am milking from the coccygeal artery/vein with a Vacutainer-EDTA (Becton Dickinson Inc, Franklin Lakes, NJ), immediately placed on ice, centrifuged (2000×g at 4°C for 10 min) and plasma samples were stored at −40°C.

### Standards Preparation

The absolute quantification of choline and choline metabolites at each WOL was determined using isotope dilution MS with the exception of GPCho where an external calibration curve was used because no commercial internal standard (IS) was available. Stock solutions of the IS in methanol were prepared so that the final concentration in the autosampler vial was *ca.* 5 times greater than the lower limit of linearity of the target compound to ensure an analyte to IS signal ratio greater than 10∶1 to avoid suppressing the target compound signal [23]. The following standards were used: Cho chloride-trimethyl-d9 (Cho-d9, Cambridge Isotope Laboratories DLM 549-1, Tewksbury, MA), Bet (Bet-d11 Cambridge Isotope Laboratories DLM 407, Tewksbury, MA), PCho chloride (PCho-d9, Cambridge Isotope Laboratories, DLM-298, Tewksbury, MA) PC-d9 (Avanti polar lipids, 860362, Alabaster, AL), SM-d13-c13 (Ricerca-custom made, Painesville, OH) ACho-d13(C/D/N Isotopes ICN D-1780, Quebec, Ca), LPC-D3 (Larodan 71-2826, Malmo, Sweden). The ratio between the area of the target compound and area of the IS was multiplied by the known IS concentration to quantify the various phospholipids. A calibration curve was used to calculate the absolute quantitation of GPCho concentration (*1-O-Octadecyl-2-O-methyl-sn-glycero-3-phosphorylcholine*, Sigma, 09262). Serial dilutions were made from 0.0012 µl to 11857.55 µl concentration of GPCho in 10 mL of methanol resulting in a linear equation using for quantification.

### Sample Preparation

Milk samples from consecutive milkings (pm, am) on each sampling day were composited proportional to milk yield at each milking prior to extraction for choline analysis. Plasma, milk and feed samples were extracted based on the Bligh and Dyer [28] method with modifications described by Zhao et al. [23]. The samples were kept on ice throughout the extraction process. Briefly, 1 ml of extraction solvent (chloroform, methanol, water, 1∶2∶0.8) was added to 200 µL of plasma or milk, or 100 mg of feed, and 40 µl of the internal standard solution. The samples were centrifuged at 28620×g and 4°C for 5 min. The resultant supernatant was transferred into a separate glass vial. The extraction procedure was repeated twice and each time the supernatants were collected into the same vial. The combined extracts were dried under constant nitrogen steam and then re-dissolved in 5 ml of methanol.

### HILIC LC-MS/MS Method

Metabolites of choline were analyzed (Figure 1) using HILIC LC-MS/MS based on methods outlined by Zhao et al. [23]. A Finnigan Surveyor MS Pump Plus coupled to a Finnigan Surveyor Autosampler was used to introduce the samples onto an Ascentis Express HILIC column (150×2.1 mm, 2.7 µm particles) HPLC separation. The autosampler tray temperature was 4°C and full loop injections of 10 µL were used. The column temperature was set at 25°C and 200 µL/min flow rate was used. The mobile phases were acetonitrile (ACN, solvent A) and 10 mM ammonium formate in water buffered to pH 3.0 with formic acid (solvent B). A 30 min gradient was performed: t) 0.1 min, 8% solvent B; t) 10 min, 30% solvent B; t) 15 min, 70% solvent B; t) 18 min, 70% solvent B; t) 18.01 min, 8% solvent B; t) 30 min, 8% solvent B. At the end of analysis the column was flushed with 100% ACN for 30 min prior to storage to preserve the stationary phase and to prevent retention time shifts in subsequent analyses.

Fused silica tubing (0.10 ID×0.19 mm OD) was connected to the ion source housing of a Finnigan TSQ Quantum Discovery MAX operating in electrospray ionization (ESI) mode for sample introduction. Ion source parameters were 4500 ESI spray voltage and 290°C ion transfer capillary temperature. Nitrogen was used as a sheath gas set at 40 arbitrary units at the instrument and 100 psi from the source. Argon collision gas was set to 1.5 mTorr in the collision cell and 20 psi from the source. The selected reaction monitoring (SRM) transitions were used with the following parameters: 0.05 s scan time, 1 *m/z* scan width, and Q1 & Q3 peak widths full width at half maximum of 0.7 Da. Parent and product masses were rounded to 0.1 *m/z* unit. A complete list of masses and collision energies used were reported by Zhao et al. [23]. SRM transitions of internal standards were: PC 799.7 to 193, SM 735.6 to 188, AC 159.4 to 91.2, LPC 499.3 to 184, Cho 113.2 to 69.1, Bet 129.1 to 66.2, and PCho 193 to 125.1. Two segments were used to lower duty cycle. Segment 1 was from 0–10.8 min, and contained the SRM transitions for all PC and SM. Segment 2 was from 10.8–30 min, and contained the SRM transitions for SM, AC, LPC, Cho, Bet, GPCho, and PCho. All Finnigan instruments were operated using Xcalibur (Thermo Fisher Scientific Inc., Waltham, MA, version 2.0.7) installed on a Dell Precision 390 computer. The data was processed with the Xcalibur 2.0.7 Quan Browser software (Thermo Fisher Scientific Inc., Waltham, MA).

### Statistical analysis

Effects of WOL on milk composition and milk and plasma choline metabolites were analyzed using the GLIMMIX procedure of SAS (SAS version 9.3 Institute Inc., Cary, NC, USA). The model included WOL as fixed effect, cow as a random effect, with WOL as the repeated measure, and spatial power as the covariance structure because of unequal sampling intervals. Means are reported as least squares means with their respective pooled standard errors and considered to differ when P≤0.05. For choline metabolites that had unequal variance the data was Log10 transformed.

Hepatic and mammary PEMT activity was evaluated from the PEMT ratio which was calculated based on the ratio of PC species containing DHA to total PC in blood and milk, respectively [25], [29]. While in two instances there was coelution of PC containing DHA with other long-chain polyunsaturated fatty acids, DeLong et al. [29] reported the same coelution of PC species associated with PEMT activity. Relationships between WOL, milk and plasma choline metabolites and PEMT activity were explored. When effects were found significant, polynomial (PROC GLIMMIX) and exponential (PROC NLIN) relationships were evaluated. Model adequacy was assessed based on mean square error, residuals against both fitted and predicted values, and normal probability plot. In addition, relationships between choline metabolites in milk and blood were examined. Variable selection was performed and relationships between selected variables were evaluated using PROC REG. Finally, multiple regression analysis was conducted using PROC REG to assess the relationship between milk total choline yield and animal performance variables (i.e. milk yield and milk composition) during lactation.

## Results

Animals were sampled on each of three days during early, mid and late stages of lactation; their yield and composition of milk is shown in Table 3. As expected, milk yield followed the typical lactation curve, increasing from early to mid-lactation and then decreasing during late lactation. Milk protein yield did not change during lactation; however, milk fat yield was higher in early lactation and then decreased as lactation progressed. Milk fat and protein concentration were greater at the start of lactation, lowest at mid and intermediate at the end of lactation.

**Table 3:** Milk yield and composition of dairy cows during lactation.

	Week of lactation									
	1	2	3	7	10	13	31	34	37	SEM
Milk, kg/d	26.6^ef^	32.8^bcd^	36.0^ab^	36.9^ab^	38.2^a^	35.7^ab^	30.5^cde^	29.1^def^	24.8^f^	2.01
Protein										
%	3.88^a^	3.19^b^	2.91^c^	2.67^d^	2.77^cd^	2.75^cd^	3.22^b^	3.21^b^	3.22^b^	0.09
g/d	1020	1038	1049	983	1059	986	992	938	795	65.5
Fat										
%	5.55^a^	5.12^a^	5.00^abc^	4.20^c^	3.95^c^	3.81^c^	4.27^bc^	3.94^c^	4.37^bc^	0.29
g/d	1478^bc^	1628^ab^	1808^a^	1538^abc^	1493^bc^	1372^bcd^	1307^cd^	1146^d^	1097^d^	132.0

abcd Means within a row with different superscripts differ (P<0.05).

**Figure 2:**
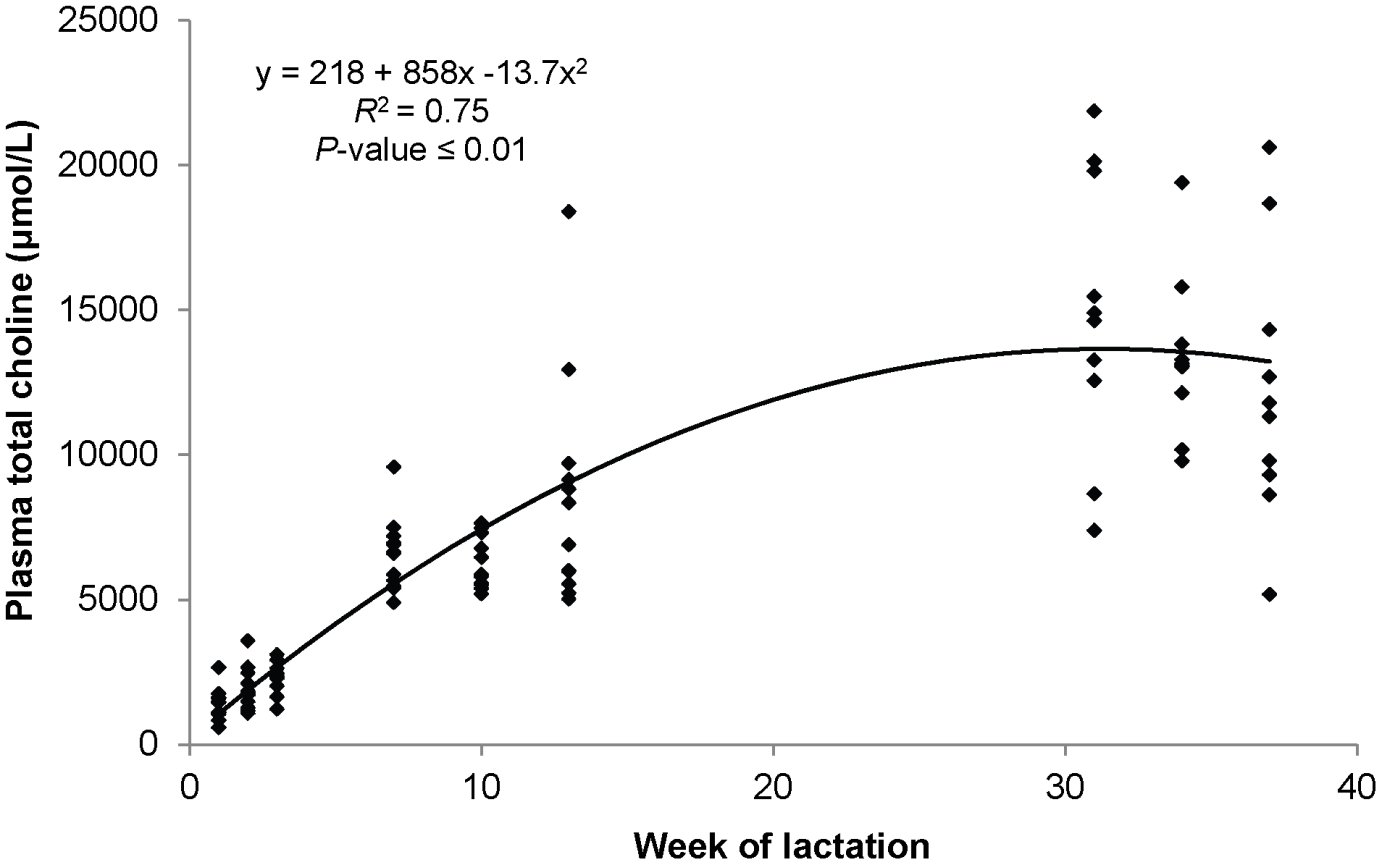
Relationship between total choline concentration in plasma and week of lactation. The standard errors for the intercept, linear and quadratic term were 564, 97.0, and 2.42, respectively.

The main choline metabolites detected in plasma were PC, SM and LPC, with the concentration of all metabolites changing over lactation (Table 4). PC was the predominant choline metabolite found in plasma, with its proportion of total choline increasing from 77% at the onset of lactation to 95% by late lactation. Also PCho and Bet concentrations in plasma were the highest at the end of the lactation. In contrast, SM and LPC concentrations in plasma increased from early to mid-lactation and then decreased in late lactation. ACho was not detected in plasma and as its internal standard was also not detected, it is likely the metabolite was degraded. The concentration of total choline in plasma showed a positive quadratic relationship with WOL (Figure 2), increasing up to 10-times from early to late of lactation, which was mainly explained by the increase in PC. The profile of fatty acids bound to LPC, containing a single fatty acid, and PC, containing a combination of two fatty acids, were quantified in plasma and milk during lactation. The plasma concentration of all forms of LPC and PC changed during lactation (Table 5). The main fatty acid associated with LPC in plasma was linoleic acid (C18:2) and all forms, except LPC containing stearic acid (C18:0), increased from early to mid-lactation and then decreased in late lactation. For PC, the concentrations of all species increased in plasma from early to late lactation regardless of fatty acyl composition.

**Table 4:** Choline and choline metabolites concentration in plasma (µmol/L) of dairy cows during lactation.

Metabolite^1^	Stage and week of lactation											
	Early				Mid				Late			
	1	2	3	SEM	7	10	13	SEM	31	34	37	SEM
Bet^2^	25.5^cd^	18.4^e^	13.7^e^	3.48	18.3^de^	18.6^de^	20.4^de^	3.48	32.0^c^	38.5^b^	48.9^a^	3.84
Cho	3.75	3.92	4.47	0.35	3.75	4.16	4.01	0.35	3.45	3.02	3.26	0.38
PCho^2^	0.43^b^	0.45^b^	0.41^b^	0.09	1.22^a^	1.28^a^	1.57^a^	0.37	1.78^a^	1.43^a^	1.66^a^	0.39
GPCho^2^	1.12^cd^	0.56^d^	1.03^b^	0.32	4.12^ab^	4.37^ab^	5.03^a^	0.98	2.36^abc^	2.46^abc^	1.78^b^	0.81
LPC	113^d^	133^d^	196^c^	24.7	321^ab^	251^c^	363^a^	25.8	250^bc^	243^c^	218^c^	30.2
SM	189^d^	231^cd^	273^c^	28.0	648^a^	593^a^	626^a^	28.0	444^b^	401^b^	404^b^	30.6
PC^2^	1014^e^	1437^d^	1757^c^	132	5515^b^	5491^b^	6803^b^	560	13535^a^	12593^a^	10894^a^	1253
TC^3^	1305^f^	1805^e^	2220^d^	159	6456^bc^	6325^c^	7866^b^	614	14241^a^	13255^a^	11536^a^	1250

1 Betaine (Bet), free choline (Cho), glycerophosphocholine (GPCho), lysophosphatidylcholine (LPC), phosphatidylcholine (PC), phosphocholine (PCho), sphingomyelin (SM), total choline (TC).

2 Variables were Log10 transformed to obtain equal variance. Means and SEM are back-transformed.

3 TC is the sum of Cho, LPC, PC, PCho, SM and GPCho.

abcd Means within a row with different superscripts differ (P<0.001).

**Table 5:** Concentration of lysophophatidylcholine (LPC) and phosphatidylcholine (PC) with a particular fatty acyl chain in plasma of dairy cows during lactation.

	Stage and week of lactation											
	Early				Mid				Late			
	1	2	3	SEM	7	10	13	SEM	31	34	37	SEM
**Metabolite concentration** 1 **, µmol/L**												
LPC												
16∶0	18.9^e^	22.7^de^	30.7^c^	2.97	52.4^a^	41.1^ab^	50.7^a^	5.07	33.2^bc^	28.9^cd^	28.0^cd^	3.51
18∶0	18.1^d^	23.7^d^	34.4^c^	4.01	64.5^ab^	53.5^b^	73.2^a^	8.53	65.2^ab^	59.1^ab^	54.1^ab^	8.29
18∶1	24.7^d^	27.9^d^	39.6^cd^	5.87	62.5^a^	50.3^abc^	59.0^ab^	5.87	48.4^abc^	41.7^bcd^	38.6^cd^	6.46
18∶2	40.2^f^	56.0^e^	77.2^d^	9.6	161.9^a^	125.7^ab^	165.3^a^	20.5	113.7^bc^	99.4^bcd^	86.6^cd^	15.4
PC												
16∶0/16∶0	8^d^	12^c^	13^c^	1.5	53^b^	52^b^	65^b^	7.5	166^a^	140^a^	127^a^	20.7
16∶0/16∶1	8^d^	10^c^	12^c^	1.4	44^b^	42^b^	52^b^	6.1	132^a^	114^a^	105^b^	16.7
16∶0/18∶1	142^d^	183^c^	210^c^	19.7	497^b^	476^c^	534^b^	50.2	970^a^	901^a^	851^a^	97.8
16∶0/18∶2	265^f^	371^e^	457^d^	42	1423^c^	1431^d^	1694^c^	156	3160^a^	2896^ab^	2413^b^	315
16∶0/20∶3	86^f^	119^e^	148^d^	14	435^c^	459^c^	577^b^	53	1046^a^	1009^a^	844^a^	104
16∶0/20∶4	34^e^	50^d^	64^c^	6.4	204^b^	203^b^	253^b^	25.3	497^a^	445^a^	394^a^	53.7
16∶0/20∶5,16∶1/20∶4^2^	7^e^	9^d^	11^e^	1.3	40^b^	38^b^	50^b^	5.6	120^a^	111^a^	102^a^	14.7
16∶0/22∶6,18∶1/20∶5,												
18∶2/20∶4^2^	6.8^e^	9.3^d^	10.7^d^	1.1	36.1^c^	33.4^c^	41.8^c^	4.4	94.6^a^	74.0^ab^	65.0^b^	10.9
18∶0/18∶1	107^d^	153^c^	184^c^	18.2	501^b^	493^b^	594^b^	58.8	1145^a^	1073^a^	934^a^	98.9
18∶0/18∶2,18∶1/18∶1^2^	243^g^	356^f^	446^e^	42	1394^d^	1479^d^	1856^c^	176	3385^a^	3269^ab^	2633^b^	346
18∶0/20∶3	24^f^	41^e^	58^d^	8	286^bc^	271^c^	340^b^	52	1085^a^	1050^a^	974^a^	156
18∶0/20∶4	35^e^	54^d^	67^d^	8	305^bc^	262^c^	369^b^	46	948^a^	866^a^	825^a^	128
18∶0/22∶5	12^e^	19^d^	22^d^	2.7	90^bc^	72^c^	99^b^	12.5	246^a^	209^a^	203^a^	33.8
18∶1/20∶4,18∶0/20∶5,												
16∶0/22∶5^2^	22^e^	31^d^	36^d^	4.1	125^bc^	109^c^	147^c^	16.7	348^a^	293^a^	269^b^	42.9
18∶1/22∶6	0.9^f^	1.5^e^	1.7^e^	0.20	6.6^cd^	6.0^d^	8.5^c^	1.02	19.6^a^	14.2^ab^	12.0^b^	2.56
18∶0/22∶6,18∶1/22∶5^2^	6^f^	9^e^	10^e^	1.2	42^cd^	35^d^	49^c^	5.9	121^a^	87^ab^	71^b^	16.0
PEMT Ratio^3^	0.013^c^	0.014^bc^	0.013^c^	0.0007	0.016^ab^	0.014^bc^	0.015^bc^	0.0008	0.018^a^	0.014^bc^	0.014^bc^	0.001

1 All metabolites, except for LPC 18∶1 were Log10 transformed to obtain equal variance. Means and SEM represents non-transformed data.

2 Two or three possible combinations of fatty acids were identified by the same product ions from HILIC LC-MS/MS.

3 Phosphatidylethanolamine N-methyltransferase (PEMT) ratio  =  (16∶0/22∶6,18∶1/20∶5,18∶2/20∶4+18∶1/22∶6+18∶0/22∶6,18∶1/22∶5)/(16∶0/16∶1+16∶0/20∶5, 16∶1/20∶4+16∶0/16∶0+16∶0/18∶2+16∶0/18∶1+16∶0/20∶4+18∶0/20∶4+18∶0/20∶3+18∶0/22∶5+18∶0/18∶2, 18∶1/18∶1+18∶0/18∶1+18∶1/20∶4,18∶0/20∶5,16∶0/22∶5).

abcdef Means within a row with different superscripts differ (P<0.001).

**Figure 3:**
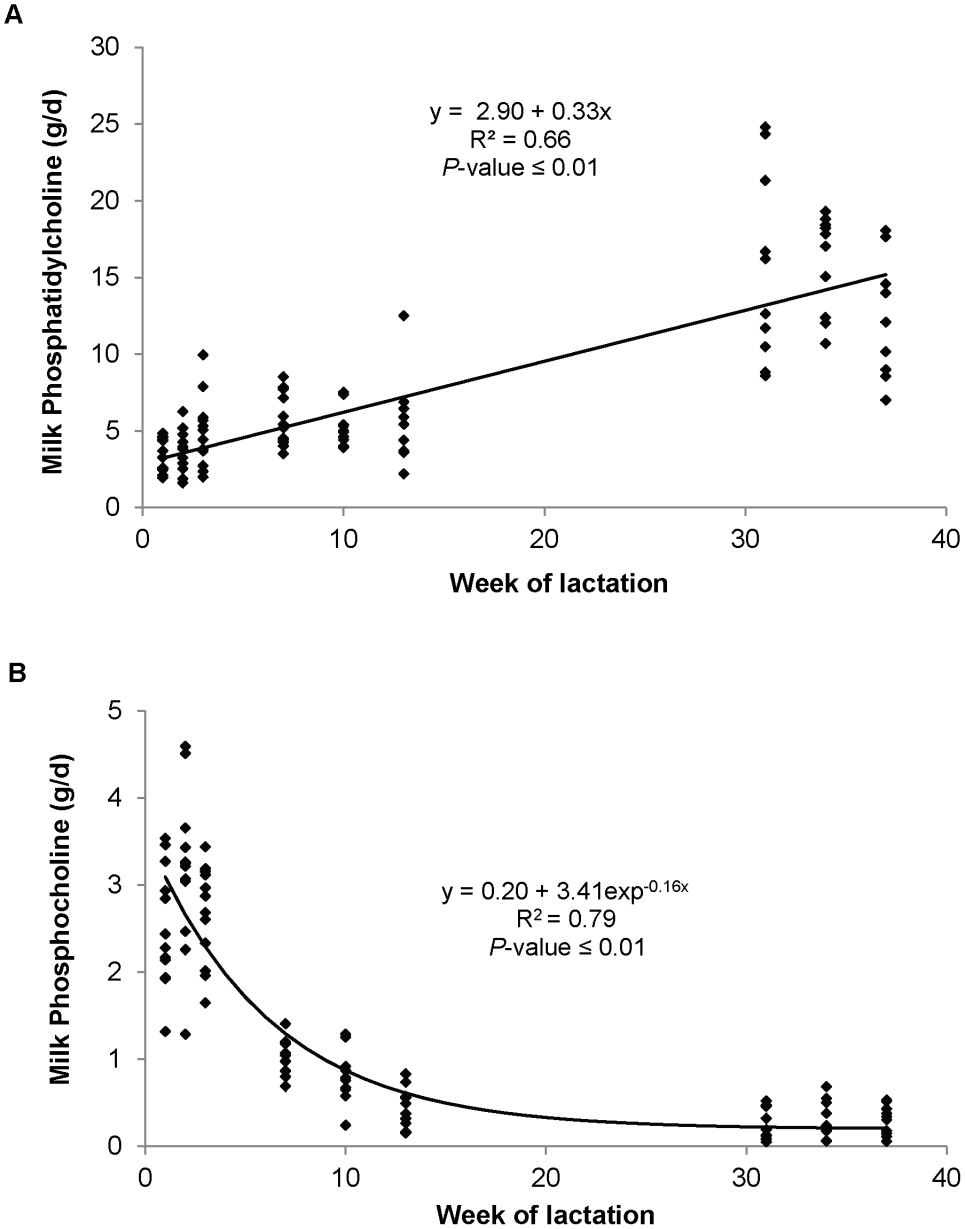
Relationship between week of lactation and milk yields of phosphatidylcholine (A), phosphocholine (B). The standard errors for the intercept and linear terms in (A) were 0.46 and 0.02, respectively. The equation was y = a+b exp^−cx^ with the standards errors for the fitted parameters in (B) were 0.106, 0.190, and 0.022 for a, b and c, respectively.

The concentration and yield of all choline and choline metabolites in milk, except for LPC yield, changed during lactation (Table 6). The two main choline metabolites in milk were PC and PCho, which together represented 60 to 80% of the total choline in the milk. The yield of PtdCho increased linearly through lactation, from <5 g/d in early lactation to >12 g/d by late lactation (Figure 3A). In contrast PCho was the main choline metabolite in early lactation (averaged 2.7 g/d), and its yield displayed an exponential decay as lactation progressed (Figure 3B).

**Table 6:** Concentration and yield of choline and choline metabolites in milk of dairy cows during lactation.

	Stage and week of lactation											
	Early				Mid				Late			
	1	2	3	SEM	7	10	13	SEM	31	34	37	SEM
**Metabolite concentration** 1 **, µmol/L**												
ACho	nd	nd	nd	-	0.05^a^	0.04^a^	0.04^a^	0.005	0.01^b^	0.01^b^	0.01^b^	0.006
Bet^2^	100^a^	45^cd^	27^e^	6.5	17^f^	18^f^	19^f^	2.2	37^d^	49^bc^	59^b^	5.6
Cho	28^d^	43^cd^	57^c^	11.1	101^b^	112^b^	117^ab^	11.1	118^ab^	127^ab^	144^a^	12.2
PCho^2^	527^a^	537^a^	413^b^	76.1	155^c^	113^d^	64^ef^	17.1	34^g^	42^fg^	52^e^	7.6
GPCho	102^b^	82^bc^	76^cd^	9.9	55^de^	55^de^	48^e^	10.6	156^a^	146^a^	156^a^	10.7
LPC	2.27^cd^	2.40^bcd^	2.45^bc^	0.25	1.85^d^	1.86^d^	1.75^d^	0.25	3.03^ab^	3.24^a^	3.41^a^	0.27
SM	52.8^a^	44.9^ab^	45.9^b^	2.12	33.8^c^	30.7^cd^	27.8^d^	2.12	35.8^c^	36.7^c^	36.7^c^	2.31
PC^2^	170^bc^	149^c^	167^bc^	12.7	202^b^	178^bc^	183^bc^	14.7	582^a^	701^a^	694^a^	60.8
TC^3^	908^a^	872^a^	787^b^	56.5	559^c^	501^cd^	450^d^	56.5	886^a^	1002^a^	972^a^	61.9
**Metabolite yield** 1 **, g/day**												
ACho	nd	nd	nd	-	0.0003^a^	0.0003^a^	0.0003^a^	0.00004	0.00004^b^	0.00003^b^	0.00002^b^	0.00004
Bet^2^	0.26^a^	0.16^b^	0.10^cde^	0.03	0.07^d^	0.08^d^	0.08^d^	0.01	0.12^bc^	0.16^b^	0.15^b^	0.02
Cho	0.07^d^	0.13^c^	0.20^b^	0.04	0.37^a^	0.43^a^	0.41^a^	0.04	0.36^a^	0.36^a^	0.34^a^	0.04
PCho^2^	2.43^b^	3.03^a^	2.61^b^	0.53	1.00^c^	0.75^d^	0.40^d^	0.17	0.18^f^	0.21^f^	0.24^f^	0.04
GPCho^2^	0.59^b^	0.62^b^	0.63^b^	0.08	0.47^bc^	0.49^bc^	0.40^c^	0.07	1.08^a^	0.98^a^	0.89^bc^	0.15
LPC	0.03	0.05	0.04	0.007	0.03	0.04	0.03	0.007	0.05	0.05	0.05	0.008
SM	0.95^abc^	0.99^ab^	1.13^a^	0.07	0.85^bcd^	0.78^cde^	0.68^de^	0.07	0.75^de^	0.71^de^	0.61^e^	0.07
PC^2^	3.3^d^	3.5^cd^	4.4^bc^	0.46	5.5^b^	5.0^b^	4.8^b^	0.58	14.9^a^	16.5^a^	12.1^a^	1.92
TC^2^^,^ 3	7.5^d^	8.4^cd^	9.3^c^	0.80	8.3^cd^	7.4^cd^	6.7^d^	0.64	17.5^ab^	19.0^a^	14.3^bc^	1.79
TCM^2^^,^ 4	2.35^cd^	2.79^ab^	2.81^ab^	0.21	2.01^de^	1.89^e^	1.60^f^	1.21	3.23^ab^	3.45^a^	2.72^bc^	0.29

1 Acetylcholine (ACho), betaine (Bet), free choline (Cho), glycerophosphocholine (GPCho), lysophophatidylcholine (LPC), not detected (nd), phosphatidylcholine (PC), phosphocholine (PCho), sphingomyelin (SM), total choline (TC), total choline moiety (TCM).

2 Variables were Log10 transformed to obtain equal variance. Means and SEM are back-transformed.

3 TC is the sum of ACho, Cho, LPC, PC, PCho, SM and GPCho.

4 TCM is the sum of choline moiety originating from ACho, Cho, LPC, PC, PCho, SM and GPCho.

abcd Means within a row with different superscripts differ (P<0.001).

The total output of choline in milk (based on the moiety alone) was lowest in mid lactation, averaging 1.8 g/d, whereas it averaged 2.7 g/d and 3.1 g/d in early and late lactation, respectively (Table 6). The yield of free choline in milk increased during the initial 3 weeks of lactation and then remained constant until the end of lactation (Table 6). In milk, except for 18∶0/22∶5 PC, the concentrations of all double fatty acid pairings within PC increased from early to late lactation (Table 7). The main fatty acids found in LPC were palmitic acid whereas palmitic and oleic acid together were the main combination found in PC.

**Table 7:** Concentration of lysophophatidylcholine (LPC) and phosphatidylcholine (PC) with a particular fatty acyl chain in milk of dairy cows during lactation.

	Stage and week of lactation												
	Early				Mid				Late				
	1	2	3	SEM	7	10	13	SEM		31	34	37	SEM
**Metabolite concentration** 1 **, µmol/L**													
LPC													
16∶0	0.81^bc^	0.73^c^	0.88^b^	0.11	0.87^bc^	0.84^bc^	0.90^bc^	0.11	1.49^a^		1.71^a^	1.72^a^	0.25
18∶0	0.32	0.29	0.39	0.08	0.31	0.42	0.41	0.07	0.59		0.69	0.68	0.08
18∶1	0.64^ab^	0.79^ab^	0.90^ab^	0.16	0.56^bc^	0.37^cd^	0.33^d^	0.10	0.67^ab^		0.77^ab^	0.97^a^	0.19
18∶2	0.27	0.37	0.44	0.09	0.36	0.22	0.23	0.08	0.34		0.28	0.43	0.10
PC													
16∶0/16∶0	20^c^	15^d^	21^c^	3.4	30^b^	26^bc^	28^bc^	5.0	148^a^		150^a^	108^a^	27.5
16∶0/16∶1	5.1^d^	4.2^d^	5.5^d^	0.67	8.6^c^	7.5^c^	8.9^c^	1.08	50.7^ab^		55.8^a^	38.9^b^	7.23
16∶0/18∶1	48^cd^	39^d^	50^c^	7.6	65^b^	50^cd^	56^cd^	10.2	294^a^		316^a^	205^a^	55.3
16∶0/18∶2	18^b^	14^b^	20^b^	3.2	27^b^	22^bc^	27^b^	4.6	113^a^		113^a^	75^a^	21.0
16∶0/20∶3	14.7^cd^	12.3^d^	15.9^c^	2.6	19.2^c^	14.1^cd^	16.1^cd^	3.1	58.2^a^		57.0^a^	37.5^b^	10.3
16∶0/20∶4	4.2^c^	3.3^d^	4.5^c^	0.74	5.5^c^	4.0^cd^	4.7^cd^	0.90	17.2^ab^		17.6^a^	11.3^b^	3.19
16∶0/20∶5,16∶1/20∶4^2^	1.5^b^	1.5^b^	1.8^b^	0.02	1.6^b^	1.5^b^	1.6^b^	0.02	5.2^a^		6.0^a^	4.4^b^	0.76
16∶0/22∶6,18∶1/20∶5,													
18∶2/20∶4^2^	0.61^b^	0.39^c^	0.53^b^	0.09	0.51^bc^	0.43^bc^	0.52^bc^	0.08	2.20^a^		1.28^a^	1.54^a^	0.39
18∶0/18∶1	17.0^b^	14.0^b^	17.0^b^	2.8	19.7^b^	14.7^b^	15.6^b^	3.2	62.6^a^		64.7^a^	43.7^a^	11.7
18∶0/18∶2,18∶1/18∶1^2^	31^c^	27^c^	33^c^	5.3	37^c^	27^c^	29^c^	6.0	112^ab^		111^a^	72^b^	20.1
18∶0/20∶3	0.84^b^	0.50^c^	0.69^bc^	0.14	0.97^b^	0.80^b^	0.94^b^	0.16	3.70^a^		3.80^a^	2.77^a^	0.69
18∶0/20∶4	1.74^b^	1.07^c^	1.44^bc^	0.24	1.79^b^	1.43^bc^	1.63^bc^	0.27	6.71^a^		6.82^a^	4.81^a^	1.25
18∶0/22∶5	0.68^a^	0.45^bc^	0.63^a^	0.13	0.36^c^	0.30^c^	0.34^c^	0.06	0.92^a^		0.90^a^	0.64^bc^	0.19
18∶1/20∶4,18∶0/20∶5,													
16∶0/22∶5^2^	2.89^bc^	1.93^d^	2.84^bc^	0.46	2.06^cd^	1.69^d^	1.97^cd^	0.33	6.75^a^		6.52^a^	4.63^ab^	1.19
18∶1/22∶6	0.26^bc^	0.21^bc^	0.25^bc^	0.05	0.25^bc^	0.19^c^	0.25^bc^	0.05	0.75^a^		0.67^a^	0.39^b^	0.17
18∶0/22∶6,18∶1/22∶5^2^	0.55^bc^	0.46^bc^	0.50^bc^	0.11	0.40^bc^	0.34^c^	0.43^bc^	0.08	1.14^a^		1.10^a^	0.70^ab^	0.33
PEMT ratio^3^	0.009^a^	0.009^a^	0.008^b^	0.0006	0.006^cde^	0.007^bc^	0.005^de^	0.0006	0.005^de^		0.004^e^	0.004^e^	0.0007

1 All variables, except for LPC 18∶0 and phosphatidylethanolamine N-methyltransferase (PEMT) ratio, were Log10 transformed to obtain equal variance. Means and SEM represents non-transformed data.

2 Two or three possible combination of fatty acids were identified by the same product ions from HILIC LC-MS/MS.

3 PEMT ratio =  (16∶0/22∶6,18∶1/20∶5,18∶2/20∶4+18∶1/22∶6+18∶0/22∶6,18∶1/22∶5)/(16∶0/16∶1+16∶0/20∶5, 16∶1/20∶4+16∶0/16∶0+16∶0/18∶2+16∶0/18∶1+16∶0/20∶4+18∶0/20∶4+18∶0/20∶3+18∶0/22∶5+18∶0/18∶2, 18∶1/18∶1+18∶0/18∶1+18∶1/20∶4,18∶0/20∶5,16∶0/22∶5).

abcde Means within a row with different superscripts differ (P<0.001).

**Figure 4:**
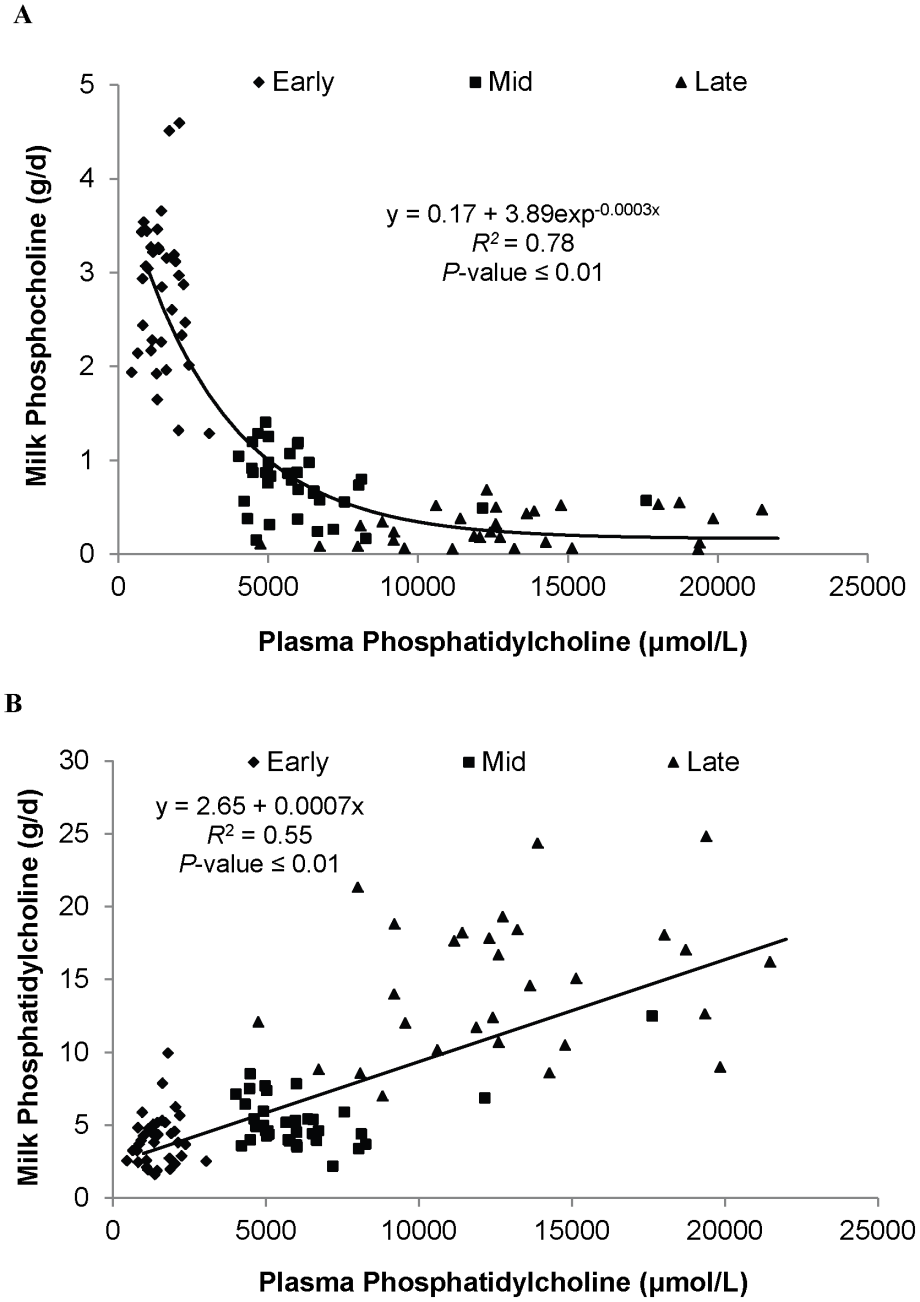
Relationship between plasma phosphatidylcholine concentration and either yield of phosphatidylcholine (A) or phosphocholine (B). Each symbol represents either early ( ♦), mid (▪) and late (▴) lactation. The standards errors for the intercept and linear terms in (A) were 0.58 and 0.00007. The equation was y = a+b exp^−cx^ with the standard errors for the fitted parameters in (B) were 0.133, 0.230, and 0.000046 for a, b and c, respectively.

When evaluating relationships between the choline metabolites in plasma and milk we found that milk PC yield increased linearly as PC concentration in plasma increased (Figure 4A). In contrast, milk PCho yield showed a negative exponential decay with increased PC concentration in plasma (Figure 4B). We also evaluated the relationship between WOL and the PEMT ratio in plasma and milk. There was no consistent pattern in the plasma PEMT ratio over lactation, whereas in milk the ratio was highest during early lactation.

Finally, the multiple regression analysis found that WOL, milk yield and the content of fat and protein in milk, were able to explain a moderate proportion of the variation in total choline in milk during lactation (*R* ^2^ = 0.67; p<0.001). The model obtained for total choline yield in milk (γ; g/d) was
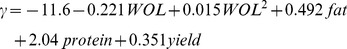



Where WOL is week of lactation, fat is percentage of fat in milk, protein is the percentage of protein in milk, and yield is daily milk yield (kg); SEE were 4.88, 0.166, 0.004, 1.24, 0.377, 0.048, respectively.

## Discussion

Milk and dairy products derived from the bovine are a rich source of choline for the human diet; however; little is known about how choline varies in milk during lactation in the dairy cow. In addition, choline is found in various water- and lipid-soluble forms in milk, which can differ in their absorption, transport and metabolism in the nursing infant [30]. When comparing the concentration of serum choline metabolites in infants fed either breast milk or bovine-based infant formula, Ilcol et al. [18] reported around 2-fold greater serum Cho levels in breast milk fed infants, with these levels positively correlated with the concentrations of water-soluble choline metabolites (i.e. Cho, PCho and GPCho) in breast milk. Therefore, characterizing the changes in choline metabolites in bovine milk during the course of lactation may form the basis to enhance the choline metabolite profile in bovine-derived infant formula. We adopted methodology that has been recently developed and validated to detect the major choline metabolites in egg yolk [23], [24]. This technique utilizes HILIC LC-MS/MS that, as well as providing improved sensitivity, has an advantage over other analytical methods as it identifies all the major choline metabolites (Figure 1) and differentiates the fatty acid chains in PC and LPC in a single chromatographic run. We also used matching IS for each analyte class, except GPCho, for absolute quantification for a member of each choline metabolite class as well as for enhanced quantification of all other compounds.

Choline and choline metabolites in blood were of interest as it is unknown how their concentrations may change during lactation in the dairy cow. When comparing free choline concentration in plasma, dairy cows in our study had 3- to 4-fold lower levels than previously reported for breastfeeding woman [18]. In ruminants dietary choline is extensively degraded in the rumen [10], [11], which may explain the low and constant levels of free choline in plasma during lactation in the present study. We did find an increase of over 10-times in the total choline concentration in plasma from early to late lactation, with the majority of the increase coming from elevated PC levels. The plasma PC concentrations in early lactation were similar to that previously reported in breast feeding women [18]; however, levels in blood were much higher at mid and late lactation in our study. PC is the main phospholipid component in all the lipoprotein classes, with its proportion ranging from 60 to 80% of total phospholipids [31]. As lactation progresses there is an increase in lipoprotein concentration in blood, primarily in HDL concentration in dairy cows [32]. Therefore, the increased concentrations of PC are expected to have been associated with the changes in types and levels of lipoproteins in plasma that occur during lactation.

The levels of the choline moiety in milk during lactation were important to consider as milk from the bovine is an important source of choline for the human diet. The USDA database for choline content in foods indicates that retail milk with 2% fat has 16 mg/100 g [14]. In the present study the total choline moiety concentration in milk was lower than that reported by USDA throughout lactation; with mid lactation levels less than half of late lactation levels (averaged 8.3, 5.0 and 11.1 mg/100 g in early, mid and late lactation, respectively). Similar choline moiety levels in milk of dairy cows, when not supplemented with choline, have previously been reported by Deuchler et al. [33] across 3 experiments (ranged from 6.6 to 8.9 mg/100 g) and by Pinotti et al. [34] (10.0 mg/100 g). The basis for the difference between these animal studies which have analyzed choline in milk collected at harvest and the USDA database is not clear, although it may relate to milk processing and/or the low number of retail samples (n = 2) used to develop the USDA database estimate. Considering this large difference in choline levels in milk and the importance of choline originating from dairy to the human population, further evaluation of choline levels in milk may be warranted to ensure choline intake meets requirements.

Although choline is considered an essential nutrient and dietary requirements have been developed for almost all domestic animal species, presently there is no requirement established for lactating dairy cows by the NRC [27]. Nutrient requirements for lactating dairy cows are partitioned by the NRC based on their use for maintenance, pregnancy, growth and lactation. Therefore one important step in developing requirements for dairy cattle is increasing the understanding of the incorporation of choline in milk and how this varies through lactation. This approach has been used in the past for humans, where the dietary reference intake for choline is 30% higher (125 mg/d) for breastfeeding women than it is for non-breastfeeding woman, with the higher level of intake calculated based on predicted levels of choline secreted daily in milk [35]. In the current study, the yield of the choline moiety increased over 2-fold from mid to late lactation (1.6 vs 3.5 g/d, respectively). Previously Deuchler et al. [33] reported choline levels in milk of cows, also when not supplemented choline, in early lactation (2.6 g/d) and mid-lactation (2.1 g/d) that is within the range observed in our study. Although these dairy cattle studies may give an indication of the choline requirement for lactation, research is needed to better understand what levels of choline are needed for non-lactating functions. This is particularly important in the case of the peripaturient dairy cow where there is a high prevalence of fatty liver disease [12], which is the classic symptom of choline deficiency [36].

Changes in choline and its metabolites during lactation have previously been reported in human and porcine milk [18], [22]; however, there has not been a systematic evaluation of changes during lactation in choline metabolites in milk of dairy cows. Recently Klein et al. [37] used NMR to provide relative quantification to estimate the range in concentration of four water-soluble choline metabolites in milk during lactation of dairy cows. For Cho, Bet and GPCho, the lowest level of the range (10% quantile) reported by Klein et al. [37] were all greater throughout lactation than the average concentrations measured in our study, however, the changes in these 3 choline forms and PCho through lactation were all similar across the two studies. When comparing the concentration of all eight choline metabolites we found that PCho was the main choline metabolite during early lactation (averaged 58% of all choline forms), whereas PC was the main choline metabolite after early lactation and increased, as a percentage of total choline forms, from 36% at week 7 to 71% at week 37 of lactation. The PC concentration in cow's milk in late lactation (ranging from 582 to 701 µmol/L) was much greater than that previously reported by Ilcol et al. [18] in human milk at any point in lactation (ranging from 97 to 155 µmol/L). In contrast, the concentration of SM in cow's milk (38 µmol/L) was lower than found in human milk (92 µmol/L) when compared across lactation [18]. In the present study, PCho was the main water soluble choline metabolite in cow's milk, which is similar that reported for porcine milk [22] and human milk [18]. In contrast to our study, Ilcol et al. [18] found no relationship between the concentration of PCho in breast milk and day after birth, although their study was cross-sectional and did not sample individuals over time. Early research comparing the choline metabolites in breast milk and infant formula suggested that infant formula had lower levels of PCho than breast milk [19], [38]; however, recent data from Ilcol [18] and the present study results indicate that there is a large range in PCho in both breast milk (100 to 1200 µmol/L) and unprocessed bovine milk (12 to 820 µmol/L). This suggests that, if an ideal PCho level can be determined for infant nutrition, milk from dairy cows in specific stages of lactation might be used to manufacture infant formula to better match breast milk concentrations of PCho.

The levels of the choline metabolites in milk are of interest as it has been shown that the absorption and bioavailability can differ across the various metabolites. Cheng et al. [20] reported that when radiolabeled choline forms were added to infant formula fed to suckling rat pups, Cho and PCho rapidly appeared in blood and the liver, whereas PC took longer to appear in blood and was metabolized differently by the liver. The different appearance rates of label for choline and PCho versus PC in blood were likely due to independent mechanisms of absorption at the small intestine [20]. PC is hydrolyzed by phospholipase A2 in the intestine and the LPC that is generated is absorbed by passive diffusion. However, PCho is hydrolyzed by phosphatase to free choline, which is absorbed via carrier-mediated transport at the intestine [36]. In addition, in the calf after birth intestinal phosphatase activity is high [39] whereas phospholipase A2 enzyme activity is low at birth and increases after weaning [40]. Therefore, the functional importance of higher milk PCho in early lactation, observed in similar concentrations (∼500 µmol/L) in the human, porcine and bovine, may be to provide a rapidly bioavailable supply of choline for sustaining growth and maintenance of new born animals.

There is little known about the uptake of choline and its metabolites from circulation by the mammary gland. In the human free choline can be taken up from circulation (via active transport) by the mammary epithelium across a steep concentration gradient [30], [41], and recently Ilcol [18] found a positive correlation between serum free choline and milk free choline concentrations (r = 0.74, p<0.01). In the current study, we found a positive linear relationship between PC concentration in plasma and PC yield in milk of dairy cows (r = 0.55, p<0.01; Figure 4A). However, unlike the trends observed in humans, we did not find an association between plasma free choline and choline in milk, which may relate to the low concentration of free choline in blood of the dairy cows throughout lactation (<4.5 µmol/L) compared to that reported in breast-feeding woman (>14.6 µmol/L).

PC can be synthesized in the body through phosphorylation of choline from the diet or via endogenous synthesis mediated by PEMT in the liver [8] and mammary gland [41]. These two pathways of PC synthesis result in different pools of bound fatty acids, with PEMT the main origin of long-chain polyunsaturated fatty acids, primarily DHA, in rodent and human hepatocytes [26], [29]. In addition, a recent study in humans [25] found that the ratio of DHA to total fatty acids in plasma PC was a useful marker for in vivo hepatic PEMT activity. Although we were not able to quantify total DHA in PC, due to coelution of some of the PC molecular species, the detection of DHA occurred in combination with other long-chain polyunsaturated fatty acids (arachidonic acid and docosapentaenoic acid) that originate primarily from the PEMT pathway [26], [29]. We did not find a consistent pattern in the PEMT ratio in plasma across lactation, but a reduction in the ratio in milk from 0.009 to 0.004 was observed from early lactation to late lactation. Although the PEMT ratio has not been used to predict PEMT activity in the mammary gland, our results may suggest that mammary endogenous biosynthesis of choline dropped from early to late lactation and this was consistent with increasing plasma PC concentrations as lactation progressed. Interestingly, the plasma PEMT ratio in the current study was several-times lower than that reported by da Costa et al. [25] and Pynn et al. [26] in plasma of humans and mice, suggesting PC derived from hepatic PEMT activity may contribute a smaller proportion of total PC in the cow. This aligns with earlier results reported for ruminants by Robinson et al. [42] where they found hepatic PEMT in vitro activity from sheep tissues was around one-quarter of that measured in the rat. In addition, unlike rodents and humans, where almost all PEMT activity occurs at the liver [8], Robinson et al [42] reported that the majority of choline derived from PEMT activity came from extrahepatic tissue in sheep, with skeletal muscle contributing around 60% of this activity.

Multiple regression analysis indicated that milk production variables (i.e., fat and protein content and yield of milk), along with WOL, were associated with the total amount of choline secreted in milk. This result suggests that choline metabolites in milk, in addition to being located in the bilayer membrane of the fat globule [43], may also be related to the protein component in milk. As a greater understanding of the requirement for choline is developed for the lactating dairy cow there may be value in predicting milk choline output based on easily measurable variables on farm. The association we found in this study, although developed based on a small sample set of cows, suggest that the prediction of milk choline output may be possible with typically measured animal and production variables.

In conclusion, the present study quantifies the eight major choline metabolites in blood and milk during lactation in the dairy cow. Total choline in plasma increased over 10-times from early to late lactation, with the majority of the increase coming from elevated PC levels. The concentration of the choline moiety in milk decreased from early to mid-lactation before rising again in late lactation; however, throughout lactation milk choline remained below the levels reported previously in commercial milk. In milk PCho was the main metabolite in early lactation and decreased rapidly over the first weeks of lactation. The high PCho levels in early lactation have also been seen in the human and porcine and may be of importance in ensuring a rapidly bioavailable form of choline for the nursing young. In contrast, PC in milk was lowest in early lactation and increased steadily through lactation, with this change positively correlated with PC in plasma. Finally, we found that production variables (milk yield and milk fat and protein content) along with WOL were correlated with total milk choline yield, suggesting that it may be possible to predict milk choline output from easily measureable variables on the farm.
